# Assessing the Prevalence of School Burnout in German High Schools: Psychometric Properties, Gender Differences, and Cut-Off Criteria

**DOI:** 10.3390/ejihpe14060120

**Published:** 2024-06-20

**Authors:** Diana Schaefer, Kirsten Schuchardt, Claudia Maehler

**Affiliations:** Department of Educational Psychology and Diagnostics, University of Hildesheim, 31141 Hildesheim, Germany; schuchar@uni-hildesheim.de (K.S.); maehler@uni-hildesheim.de (C.M.)

**Keywords:** school burnout, prevalence assessment, MBI-SuS psychometrics, gender and grade level differences, cut-off criteria comparison

## Abstract

School-related stress and burnout can have serious consequences for students’ well-being and academic outcomes. However, there are few studies that assess the prevalence of school burnout, especially in Germany. The present study aims to determine the percentage of *N* = 1117 high school students who are likely to suffer from school burnout—also with regard to differences in gender and grade level. For this purpose, two different cut-off criteria are compared. Prior to this, the psychometric quality of the MBI-SuS adapted to the school context is examined. The validity and reliability of the three-factor MBI-SuS could be confirmed. Scalar measurement invariance was found for grade level but only partially for gender. The overall prevalence of school burnout of 20.9% found with the common cut-off criterion fits international prevalences, whereas the prevalence of 4.6% (determined with our recommended content-related cut-off criterion) is in line with observations from clinical practice. Depending on the cut-off value, girls suffer slightly more from school burnout, but no differences were found with respect to grade level. Results indicate that a substantial proportion of students are at risk for school burnout, highlighting the importance of prevention and intervention. Criteria for cut-off values should be applied with caution.

## 1. Introduction

School well-being is an important cognitive and emotional base for successful learning and achievement, as it influences both the ability and willingness of students to meet their academic demands [1]. However, well-being appears to decline over the school years [2], while the experience of stress, especially among female students, and school burnout increase [3,4]. In this context, the greatest school stress is reported at academic track schools [5] and is characterized by high demands, pressure to perform, difficult social interactions, and worries about the future as the most significant stressors [4,6]. This chronic school stress may increase the risk of developing burnout, since school can also be seen as a working place for students [7]. So meanwhile, the concept of burnout, which was originally defined as a work-related phenomenon, has shifted from adults to adolescents and children who also show the typical three main symptoms of burnout: exhaustion, cynicism, and reduced efficacy. School exhaustion is the main symptom and can be described as chronic fatigue due to being overwhelmed by schoolwork. School-related cynicism shows up in an indifferent or detached attitude toward schoolwork, loss of interest, and finding school less meaningful. Reduced academic effectiveness, also known as sense of inadequacy, refers to a diminished sense of competence and less successful performance [8]. Additionally, many students with burnout symptoms report psychosomatic complaints like abdominal pain, headaches, back pain, lack of concentration, and learning problems [9]. Also, empirical studies show the negative associations of school burnout with academic outcomes and well-being, e.g., lower educational aspirations [10] and achievement [1], school dropout [3], depressive symptoms [5], negative self-image, and anxiety [11]. So, burnout has been repeatedly linked to a variety of psychological and physical complaints and is therefore of high social relevance.

To assess burnout and also school burnout, a three-factor model with the aforementioned main symptoms, which is supported by the majority of studies (e.g., [8,12]), is classically used. The most popular instrument is the Maslach Burnout Inventory (MBI) [13], which exists in many different versions, e.g., for university students in a German version (MBI-SS-GV; [12]). Another popular instrument for assessing general burnout is the Tedium Measure [14], later renamed the Burnout Measure, which focuses on physical, emotional, and mental exhaustion and is therefore highly correlated with the homonymous MBI-subscale [15].

There are no official data on the prevalence of school burnout in Germany, but from clinical experience a prevalence rate of 3–5% is estimated [9]. Higher prevalence rates have been found in other countries: 6.7% in Slovenia [11], 27.6% in Italy, 14.6% in Switzerland [16], 21% in the French-speaking part of Switzerland [17], 6.9–12.4% of male and 10.3–16.9% of female students on the academic track in Finland [7], and even 12.6–84.2% in France [18]. At this point, it is important to mention that so far there are no official burnout diagnosis and no standardized cut-off values or criteria to identify students who are likely to suffer from burnout. Brenninkmeijer and VanYperen [19] recommend the empirically validated “exhaustion + 1” criterion for research in non-clinical populations to distinguish between people high and low in burnout. An individual is classified as burnt out if his or her mean score for the exhaustion subscale is high and additionally the person reports high depersonalization/cynicism or low personal achievement/efficacy. In their study, “high values” mean scoring in the 75th percentile or higher and “low values” scoring in the 25th percentile or lower. Some studies proceeded the same way (e.g., [20]) but others (e.g., [11,17]) used a tercile solution (values upper/lower the 33rd percentile), which is an even more liberal criterion denoting more individuals as burnt out. Thus, assignment to the “burnt out” group in these studies was not based on clinical or content-related criteria but on the basis of the distribution of measured values. Thus, it may be questionable whether the upper third of the individuals studied are really burned out in the sense of a clinical diagnosis. Other studies attempted to circumvent this problem by specifying content-related criteria for classifying a subscale as high. For example, Blaszcyk et al. [21] referred to “high” expressions of student burnout on the three subscales when the values were ≥4 (at least once a week) on a seven-point scale from 0 = never to 6 = daily. Moreover, different burnout instruments are used in these studies, so the values should be compared and interpreted carefully.

In terms of gender differences, there are studies to show that girls experience more school-related stress [4,6], and their self-esteem seems to depend more on their academic success than that of boys [22], so they should show more burnout than boys as different studies have already revealed (e.g., [5,11,22]), but only a few studies examined if the instruments used show measurement invariance. However, Li et al. [23] were able to prove measurement invariance of the Adolescent Student Burnout Inventory (ASBI) for girls and boys. 

Regarding differences in grade level, especially in grades eight to ten, students are often in an important transition phase. They have to adjust to more challenging content as they slowly prepare for the upper school. This phase can be particularly stressful as the educational pressure increases. At this age, adolescents are also undergoing major physical and psychological changes due to puberty. These can increase vulnerability to stress and affect mental health [24], so this could be an important developmental phase in which the risk of school burnout is particularly high. It can be assumed that school burnout rates increase with increasing grade level, as this could be found for school-related stress, too [4]. Bask and Salmela-Aro [3] showed that experiences of school burnout are increasing among students choosing the academic track, which is not surprising since school tasks become more challenging with higher class level. To better assess the relevance and development of school burnout, more (international) prevalence studies with transparent, reasonable, and comparable cut-off criteria are needed. The present paper aims to fill the gap for the German school context.

The aims of the present study are (1) to examine the psychometric properties (factorial structure, concurrent validity) of the self-adapted Maslach Burnout Inventory for Students of Gumz et al. [12] for the German school context (*Maslach Burnout Inventar–Version für Schülerinnen und Schüler*; MBI-SuS), (2) to test for measurement invariance concerning school burnout in terms of gender and grade level, (3) to present prevalence rates of school burnout in German high schools—also considering differences in gender and grade level, and (4) to compare and discuss different cut-off criteria for determining cut-off values for school burnout. The following hypotheses were formulated:

1. Based on the original Maslach Burnout Inventory concepts [12,13], a three-factor model with the intercorrelated components exhaustion, cynicism, and (reduced) efficacy are expected to fit the data well.
2. The MBI-SuS scales correlate moderately (subscales cynicism and efficacy) to highly (subscale exhaustion) with the Tedium Measure [14] to support the concurrent validity of the measurement instrument.
3. There is measurement invariance for (a) gender (girls vs. boys) and (b) grade (8th vs. 9th vs. 10th), so the instrument measures the same for all subgroups.
4. The prevalence rate of school burnout is higher for girls than for boys.
5. The prevalence rate of school burnout increases with grade level.

## 2. Materials and Methods

### 2.1. Sample

**Figure 1:**
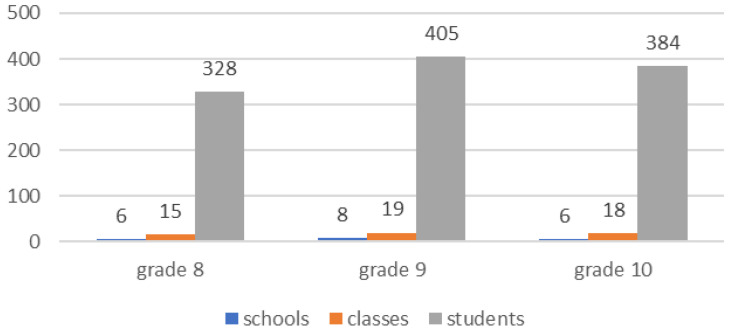
Composition of the sample depending on the class.

To obtain a sample that was as large and representative as possible for the region, all *N* = 37 public general high schools (“*Gymnasien*”) in Hanover and Hildesheim (federal state of Lower Saxony in Germany) were asked to take part in this study. Nine of them (24%) cooperated, resulting in data from *N* = 1117 students from fifty-two classes in grades eight (29.4%), nine (36.3%), and ten (34.4%) that were assessed from October 2019 to January 2020. See Figure 1 for the detailed composition. *Gymnasien* can be considered as academic track schools that prepare for university admission. At the time of the survey, students were 12 to 18 years old (*M* = 14.56, *SD* = 1.06), and 56% indicated that they were female and 43% male, which is representative for this sample [25]. They were asked to fill out a paper–pencil questionnaire during one school lesson in the presence of a teacher and a research associate. There were no specific inclusion or exclusion criteria. Students and parents were informed about the voluntariness and anonymity in this study, and written consent was obtained previously. Furthermore, permission was also granted by the Department of Education of the concerned district.

### 2.2. Measures

School burnout was assessed using the German version of the Maslach Burnout Inventory for Students (MBI-SS-GV; [12]) that was self-adapted for the school context (*Maslach Burnout Inventar–Version für Schülerinnen und Schüler*; MBI-SuS), as there was no German version of the MBI for high school students at the time this study was planned. The aim was to achieve the greatest possible similarity to the original but also appropriateness (school instead of study context) and comprehensibility for 8th–10th grade students, e.g., using age-appropriate language (for example, in item 13 we have replaced words such “*angespornt* [spurred on]” with “*motiviert* [motivated]”). See Appendix A for an overview of the adapted items. The MBI-SuS consists of three subscales with a seven-point Likert scale (from 1 = *never* to 7 = *always*). The exhaustion scale represents emotional exhaustion from schoolwork, which is the main symptom of burnout. This scale consists of five items (e.g., “*Going to school is really a burden for me*”). The cynicism subscale corresponds to a cynical and detached attitude towards school and consists of four items (e.g., *“Going to school is useless anyway”*). The subscale efficacy is recoded and represents the (reduced) ability to perform at school and has six items (e.g., *“I am good at solving the problems that arise with me at school”*). So, in contrast to the other scales, low scores here reflect high school burnout. The psychometric properties of this instrument are presented below.

Another instrument for assessing burnout is the Tedium Measure [14]. The questionnaire consists of 21 items (α = 0.93) and measures burnout in terms of physical, emotional, and mental exhaustion. The response scale also ranges from 1 = *never* to 7 = *always* on a seven-point Likert scale. At the end, a sum score is computed.

### 2.3. Statistical Analyses

All statistical analyses were conducted with IBM SPSS Statistics (version 28.0) or the Mplus statistical package (version 8.3). The Kolmogorov–Smirnov test in SPSS was used to check the normal distribution of the items before conducting the analyses. This showed that the MBI-SuS values were not normally distributed (*p* < 0.001), which was confirmed by an additional graphical inspection. That is why the following calculations were performed using the maximum likelihood robust (MLR) estimator implemented in Mplus. There were only 0.5% missing data at the item level that could be handled with full information maximum likelihood (FIML) in Mplus by default.

#### 2.3.1. Examination of the Psychometric Properties of the MBI-SuS

Because the measurement instrument for assessing school burnout was self-adapted and not yet tested, we first conducted a confirmatory factor analysis in Mplus to confirm the three-factor structure of the MBI-SuS. The analytical procedure here is oriented to the original study by Gumz and colleagues [12], so the error terms of items 8 *(“Going to school is useless anyway”*) and 9 (*“My enthusiasm for school has waned”*) of the subscale cynicism were allowed to correlate. The following fit indices were used to estimate the model fit: the χ^2^ test of model fit, root-mean-square error of approximation (RMSEA), standardized root-mean-square residual (SRMR), comparative fit index (CFI), and the Tucker–Lewis index (TLI). All indices range from 0 to 1, while values closer to 0 represent a better model fit for the RMSEA (values < 0.08: acceptable fit; <0.05: good fit) and the SRMR (values < 0.008: acceptable fit). Concerning the CFI and TLI, values closer to 1 show a better model fit (values > 0.90: acceptable fit; >0.95: good fit) (see [26]).

#### 2.3.2. Concurrent Validity

To test for concurrent validity, the correlative relationship (Pearson correlation) between the MBI-SuS dimensions and the total score of the Tedium Measure ([14]) was calculated in SPSS. A high positive correlation with the exhaustion subscale, a medium to high positive correlation with cynicism, and negative correlation with efficacy would indicate concurrent validity.

#### 2.3.3. Measurement Invariance

To ensure that the MBI-SuS measures the same for all subgroups, which is required for comparing groups, we next tested measurement invariance with respect to gender and grade level in Mplus. For this purpose, we proceeded as described by Kleinke et al. [27] and used a step-up approach, where the model constraints become successively more restrictive: (1) separate testing of the measurement model in the subgroups; (2) multiple group comparison of the measurement model (baseline model); (3) testing of metric measurement invariance; (4) testing of scalar measurement invariance. The analyses were performed separately for gender with the two subgroups female and male and for grade level with the three subgroups for grades 8, 9, and 10. If metric measurement invariance is given, the meaning of the latent constructs in both samples can be assumed to be the same. In the presence of scalar invariance, it can be assumed that there are no item-specific difficulties between the samples and that the values of the latent variable can be compared between the samples. So, we aimed for scalar measurement invariance. By the fact that the data were not normally distributed and the MLR estimator was applied, the Satorra–Bentler χ^2^ difference test was used to compare the models. We considered the rule of thumb that a change in CFI (ΔCFI) of ≤0.01 should not lead to the rejections of the null hypothesis of invariance [28], as the Satorra–Bentler χ^2^ difference test is sensitive to even small changes in large sample sizes.

#### 2.3.4. Prevalence of School Burnout and Differences in Gender and Grade

As mentioned before, there are no standardized cut-off values or criteria to identify students who are likely to suffer from burnout, and only a few studies give transparent information about cut-off criteria used. In this study, we wanted to use two different criteria to define “high” values on the school burnout subscales and compare the resulting prevalence rates: first, the common distribution-dependent 25/75th-percentile-criterion, and second, the content-related criterion—both in combination with the exhaustion + 1 criterion [19]. Since burnout is defined as “a prolonged response to chronic emotional and interpersonal stressors” [29] and the MBI-SuS response scale ranges from 1 = *never* to 7 = *always* (with 4 = *sometimes* representing the middle of the scale), we decided with regard to the content-related criterion that “high” burnout values would be ≥6 (*mostly* to *always*) for the exhaustion and the cynicism subscales and ≤2 (*never* to *almost never*) for the efficacy subscale, giving a rather strict criterion that is more appropriate to a clinically relevant phenomenon. Prevalence differences in gender (female vs. male) and grade (8th vs. 9th vs. 10th) were examined using the chi-squared test of independence in SPSS.

As this procedure does not allow for the testing of differences at subscale level or interactions between gender and grade level, additional analyses of variance were calculated with gender and grade level and their interaction as independent variables and the three burnout subscales exhaustion, cynicism, and efficacy as dependent variables in SPSS. Students who did not feel they belong to either the male or female gender were excluded due to a small total sample size of *n* = 8 (0.7%).

## 3. Results

### 3.1. Examination of the Psychometric Properties of the MBI-SuS

**Figure 2:**
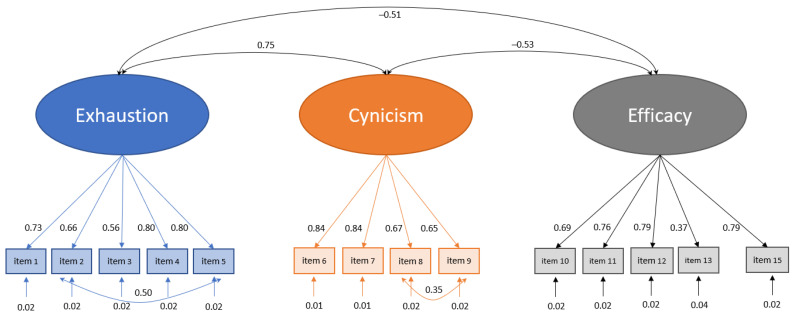
CFA of the three-factor model (standardized factor loadings, standard errors, and correlations). See Appendix A for an overview of the adapted items.

The original model, as proposed by Gumz et al. [12], did not show an optimal fit with χ^2^(86) = 795.464, *p* < 0.001, RMSEA = 0.086, CFI = 0.892, TLI = 0.868, and SRMR = 0.080. So, in the next step the model was optimized under the condition that the original three-factor model, where each item only loads on the proposed factor, is maintained. The Mplus modification indices suggested also letting the error terms of items 2 (*“At the end of a school day I feel done.”*) and 5 (*“School makes me feel exhausted”*) correlate. However, this should not indicate a misspecification of the model but rather be due to the high degree of content overlap of the items and thus not be problematic [30]. Furthermore, item 14 (*“In the course of my school years I learned many interesting things”*) did not only load on its subscale efficacy but also (negatively) on the subscale cynicism, as was found by Gumz et al. [12], too. That is why we decided to exclude this item. Adding these proposed model optimizations led to an acceptable model fit (χ^2^(72) = 452.707, *p* < 0.001, RMSEA = 0.069, CFI = 0.939, TLI = 0.923, SRMR = 0.059). All items loaded on their proposed factors. Standardized factor loadings were all significantly different from zero with *p* < 0.001, and significantly large correlations were found between all three factors (see Figure 2 for detailed results). Furthermore, the internal consistency of all subscales can be classified as good with values of Cronbach’s alpha ranging from α = 0.80 for the subscale efficacy to α = 0.85 for the subscales exhaustion and cynicism, indicating good reliability of the instrument. Thus, the three-factorial structure of the MBI-SuS with the intercorrelated components exhaustion, cynicism, and (reduced) efficacy could be confirmed. 

### 3.2. Concurrent Validity

As expected, there are consistently significant (large) correlations of the MBI-SuS subscales with the total score of the Tedium Measure. The correlation with the main dimension exhaustion is the strongest (*r* = 0.71). The cynicism dimension correlates positively (*r* = 0.55) and the efficacy subscale negatively with the Tedium Measure (*r* = −0.53). So, we could find concurrent validity for the MBI-SuS. 

### 3.3. Measurement Invariance

#### 3.3.1. Gender

We first checked the configural measurement invariance. For this purpose, the optimized model was tested separately for boys and girls. The model fits of the groups are shown in Table 1. 

The separate analyses show that the measurement model has an acceptable to good fit in each of the two groups; thus, configural measurement invariance can be assumed. In the second step, the baseline model was estimated that shows an acceptable fit, too. Then, the baseline model was compared to the more restrictive model in which the factor loadings between the groups were equated to test for metric measurement invariance. This did not lead to a significant deterioration of the model fit; therefore, metric measurement invariance can be assumed. Accordingly, we can conclude that the content of the latent constructs is the same for girls and for boys. Subsequently, it was tested whether there was a difference in the intercepts of the manifest variables between the groups (scalar measurement invariance), comparing the metric model with the scalar model. Since the model fit was significantly worse now, a full scalar measurement invariance could not be confirmed. Modification indices showed that the intercepts of five items had to be freed (items 2, 3, and 4 on subscale exhaustion and items 12 and 13 on subscale efficacy) to achieve an acceptable model fit. Since full scalar measurement invariance is rare in practice, Byrne et al. [31] propose the principle of partial measurement invariance. For meaningful comparisons of latent means, at least two manifest variables per latent variable should exhibit complete measurement invariance, which is still given in this case. Nevertheless, it is important to note that there seem to be item-specific difficulties between girls and boys in assessing school burnout, showing that girls report (only for some items) higher values when on the same burnout level.

#### 3.3.2. Grade Level

The same procedure was followed for the test of measurement invariance with respect to grade level. For model fit statistics see Table 2.

The separate analyses for the three groups show that the measurement model has an acceptable to good fit throughout; thus, configural measurement invariance is given. The baseline model also shows an acceptable fit. When comparing it to the more restrictive model with equal factor loadings between the groups, this does not lead to a significant deterioration of the model fit; therefore, metric measurement invariance can be assumed. So, the content of the latent constructs is the same for eighth, ninth, and tenth graders. Comparing the metric model with the scalar model, which tests for differences in the intercepts of the manifest variables between groups, we first see a significantly worse model fit. However, the groups differ only in one exhaustion item (item 1) and in one efficacy item (item 13), and the CFI decreases by only 0.006 units, so that it is still possible to assume scalar measurement invariance [28] and thus compare mean values between grades 8, 9, and 10.

### 3.4. Prevalence of School Burnout and Differences in Gender and Grade Level

Using the common distribution-dependent 25/75th-percentile criterion, a cut-off value of 5.4 was found for exhaustion (75th percentile), of 4.75 for cynicism, and of 4.0 for efficacy (25th percentile). This resulted in 27.8% (*n* = 310) individuals high in exhaustion, 28.3% (*n* = 315) high in cynicism, and 26.5% (*n* = 295) low in efficacy. Following the exhaustion + 1 criterion, 20.9% (*n* = 233) could be identified as at risk for burnout. Subgroup prevalence rates are shown in Table 3. The chi-squared difference test did not reveal differences in terms of grade level (χ^2^ (2) = 2.527, *p* = 0.283, *n* = 1114), but girls show a significantly higher prevalence rate of school burnout than boys using this criterion (χ^2^(1) = 4.002, *p* = 0.045, *n* = 1104) with a very small effect size of φ = 0.045.

When using the content-related criterion to determine “high” values on the school burnout subscales, results indicate that 14.2% (*n* = 158) of the students under study show high exhaustion scores (≥6), 6.9% (*n* = 77) high cynicism scores (≥6), and 1.3% (*n* = 15) severely reduced efficacy (≤2). Using the exhaustion + 1 criterion, we found a prevalence of 4.6% (*n* = 51) of students probably suffering from school burnout. Subgroup prevalence rates are shown in Table 3. No differences could be found in terms of gender (χ^2^(1) = 0.419, *p* = 0.517, *n* = 1104) or grade level (χ^2^(2) = 1.367, *p* = 0.505, *n* = 1114). It should be noted that students with high burnout values who did not feel they belong to either the male or female gender were excluded from the chi-squared test due to a small total sample size of *n* = 8 (0.7%).

The results of the analyses of variance, conducted with the whole sample, showed no significant differences between subgroups, with two exceptions: girls show higher exhaustion than boys regardless of grade level (*M*_Diff_ = 0.38, 95%-CI [0.20, 0.55], *p* < 0.001), and eighth graders exhibit lower cynicism than ninth graders (*M*_Diff_ = −0.33, 95%-CI [−0.57, −0.10], *p* = 0.002) and tenth graders (*M*_Diff_ = −0.50, 95%-CI [−0.74, −0.27], *p* < 0.001). However, there were no interaction effects between gender and grade level, neither for exhaustion nor for cynicism or efficacy.

## 4. Discussion

The aims of the present study were (1) to examine the psychometric properties of the self-adapted MBI-SuS, (2) to test for measurement invariance concerning school burnout in terms of gender and grade level, (3) to present prevalence rates of school burnout in German high schools—also considering differences in gender and grade level, and (4) to compare and discuss different cut-off criteria for determining cut-off values for school burnout: first, the common distribution-dependent criterion which defines “high” values lying in the 75th percentile or higher, and second, the stricter content-related criterion defining “high” values as values ≥6 (*mostly* to *always*) and “low” values as values ≤2 (*never* to *almost never*) on a response scale from 1 = *never* to 7 = *always*. Possible differences in gender and grade level were considered, assuming that burnout rates are higher for girls than for boys and increasing with higher grade level.

### 4.1. Interpretation of the Results

Consistent with hypothesis 1, the three-factorial structure of the MBI-SuS with the intercorrelated components exhaustion, cynicism, and (reduced) efficacy could be confirmed. Only item 14 “*In the course of my school years I learned many interesting things*” had to be excluded due to it loading negatively on the cynicism subscale and not as proposed on efficacy. This was also found by Gumz et al. [12], which is not surprising because it could be interpreted as a recoded cynicism item demonstrating a lack of interesting or important subject matter. However, the exclusion did not negatively influence the model fit. Hypothesis 2 could be confirmed too, since the three MBI-SuS subscales are consistently largely correlated with the Tedium Measure, especially (as expected) the subscale exhaustion, revealing concurrent validity. Concerning hypothesis 3, metric measurement invariance is given for gender and grade level, so the meaning of the latent constructs in all subsamples can be assumed to be the same. However, the aimed scalar measurement invariance could only be fully confirmed for the grade level. The same construct is measured, but some items seem to differ in difficulty between girls and boys: for the same level of burnout, girls give higher scores. So, burnout in boys should not be underestimated in practice. Since many studies did not test for measurement variance concerning gender, this could be one aspect to explain why some studies find differences in school burnout levels between girls and boys while others do not. Another reason for the gender differences found could be the cut-off criterion in use. In the present study, a difference could be found only using the common (distribution-dependent) criterion that revealed that girls suffer slightly more from school burnout than boys. However, this finding should be interpreted with caution, as the effect is very small and could have occurred by chance. Another point to note regarding subgroup differences is that it seems to be useful to look at school burnout at subscale levels. The analyses of variance showed, for example, that girls are significantly more exhausted than boys, regardless of the grade level. But this gender effect could not be found for the subscales cynicism and efficacy. So, Hypothesis 4 can only be supported to a limited extent. In terms of general prevalence rates, using the common distribution-dependent cut-off criterion leads to a prevalence of school burnout of 20.9%, which is in line with the prevalence rates of other European countries, e.g., 27.6% for Italy [16] and 21% for the French-speaking part of Switzerland [17]. But it could be discussed if these are the prevalence rates of students who are really burnt out in the sense of a clinical diagnosis since the values appear to be pretty high. In comparison, in 2018/2019 when these data were collected, 10.4% of young people in Germany had clinically relevant depressive symptoms [32] and about 10% of children and adolescents in Germany suffered from acute anxiety disorders before the pandemic [33]. Moreover, this criterion is highly dependent on the sample studied: if a student with high burnout scores is in a lightly stressed sample, he is more likely to be identified than if he is in a highly stressed sample. This would not happen with a criterial instead of relative reference norm. Using the content-related criterion, which is oriented towards the chronic experience of stressors leading to burnout, on the other hand, reveals a prevalence rate of 4.6% that confirms observations from clinical practice which may indicate that an adequate choice was made regarding this cut-off criterion. For example, Schulte-Markwort and Wiegand-Grefe [9] stated that 3–5% of children and adolescents suffer from school burnout with pronounced and prolonged exhaustion, which leads to reduced performance capacity and often also to physical symptoms. Even when considering this stricter cut-off criterion, 14.2% of the students under study reported high exhaustion values. This is an alarming finding, especially when considering possible time lagged effects. Exhaustion is the main symptom of burnout and the first one to become visible [5]. Some studies assume a stage model in which exhaustion leads to higher cynicism and subsequently lower efficacy (e.g., [34]), which could be interpreted in line with findings of this study showing that under the content-related criterion, 14.2% show high exhaustion, 6.9% high cynicism, and only 1.3% low efficacy. However, this should be investigated in more detail in longitudinal studies examining the development of school burnout. Concerning hypothesis 5, no significant differences in the prevalence rates were found with respect to grade level. At first sight, this contradicts the findings in the literature, including those of Gabola et al. [16], who studied adolescents of similar ages. But with regard to subscales, at least for cynicism, eighth graders show significantly lower values than ninth and tenth graders. These differences are not reflected in the prevalence rates, which could be due to the fact that cynicism is not as strongly reflected in the prevalence rates as the main symptom exhaustion. Nonetheless, it is an interesting finding that there is obviously a change in attitude towards school between grades eight and nine. Even if these students are not more exhausted during this time, something seems to change in the perceived meaningfulness of school. Further studies should take a closer look at this development, possibly combined with qualitative methods to better understand what leads to this cynical attitude.

### 4.2. Practical Implications

The practical implications of this study are limited, as it deals with the assessment and prevalence of school burnout and only demographic independent variables were included. Statements on relevant predictors are therefore not possible. However, the presented cut-off criteria could help (e.g., teachers or practitioners) to identify students who might be affected by school burnout. Furthermore, the results of this study indicate that a substantial proportion of students are at risk for school burnout or even suffer from school burnout, which can have serious consequences for students’ well-being and academic outcomes. It is therefore important that teachers, school administrators, and policymakers are aware of this problem and take it seriously. They could provide professional development for educators on how to recognize burnout symptoms, increase access to mental health services and counseling for students, and foster a supportive school environment that encourages students to express their concerns. Teachers should be aware that although there are no significant differences in the general prevalence of burnout between grades eight and ten, the perceived meaningfulness of school decreases in the transition from grade eight to nine and that this is probably an important phase in counteracting cynicism. Also, they should not underestimate burnout in boys, even if girls report symptoms more explicitly. Further studies can build on the findings of this study to evolve specific strategies that schools or educational authorities can implement to mitigate burnout, as performed by, e.g., Simonsen et al. [35].

### 4.3. Strengths and Limitations of the Study

This study is based on a rather big sample of over 1000 students with few missing data and includes the validation of the instrument used, measurement invariance testing before comparing subgroups, and transparent cut-off values. Nevertheless, some limitations have to be considered. First, the data collection took place before the COVID-19 pandemic, so a current survey is needed (also to compare pre/post-pandemic data). Second, the sample is limited to high school students (*Gymnasiast:innen*) from two cities of Lower Saxony in the grade levels eight to ten. Future studies should investigate broader age groups, different school tracks (not only academic), and countrywide samples, preferably all federal states, since they differ in their education systems, to present school burnout prevalence rates for Germany. These recommendations should also apply to other countries under study. Third, most studies, like the present one, only use self-reports to determine burnout prevalence rates. Future studies should consider external reports, e.g., from teachers, or relate prevalences determined by questionnaires to clinical impressions, such as the work carried out by Wickramasinghe et al. [36]. Last but not least, (school) burnout is not an official diagnosis in any classification system yet, so it is difficult to agree on cut-off values and what they mean exactly. That is why burnout prevalence rates in general should be treated with caution, especially when comparing prevalence rates between different countries, school systems, etc.

## 5. Conclusions

The MBI-SuS seems to be a reliable and valid instrument to assess school burnout, even showing measurement invariance for grade levels eight to ten and for gender. A considerable proportion of German high school students are at risk of burning out at school or even suffer from it, although there are no general differences in prevalence in terms of gender or grade level. Prevalence studies are important for understanding the relevance of the problem and also the extent and type of support that is needed, and that is why transparent and valid cut-off criteria should be used. What do the percentages tell us: (a) do they quantify the number of students who are highly stressed and therefore have a high risk for getting burnt out in the long run, or (b) do they indicate the number of students already burnt out in the sense of a clinical diagnosis and in need of psychotherapeutic support? We should be careful with reporting unrealistic high prevalence rates of (school) burnout since this could undermine the seriousness of this problem. So, in this paper we want to suggest that the common distribution-dependent criterion should be used to determine the prevalence of students who have a high burnout risk and the stricter content-related criterion to determine the prevalence of students who probably suffer from manifest burnout. A discourse and consensus on what is meant by school burnout and transparent criteria for capturing this phenomenon (also in an international context) are necessary.

## Figures and Tables

**Table 1:** Model fit statistics for measurement invariance concerning gender.

Model	χ^2^ (df)	*p*	CFI	TLI	RMSEA	SRMR	Δχ^2^ (df)	ΔCFI	*p*
1a: configural (female)	322.706 (72)	<0.001	0.929	0.911	0.075	0.061	-	-	-
1b: configural (male)	202.697 (72)	<0.001	0.952	0.939	0.062	0.058	-	-	-
2: baseline	525.668 (144)	<0.001	0.939	0.923	0.069	0.060	-	-	-
3: metric	537.791 (155)	<0.001	0.939	0.928	0.067	0.061	12.123 (11)	0.000	0.512
4: scalar	683.739 (166)	<0.001	0.917	0.909	0.075	0.067	145.948 (11)	−0.022	<.001
4e: partial scalar	548.974 (161)	<0.001	0.938	0.930	0.066	0.060	11.183 (6)	−0.001	0.124

χ^2^(df) = chi-squared test of model fit and its degrees of freedom; CFI = comparative fit index; TLI = Tucker–Lewis index; RMSEA = root-mean-square error of approximation; SRMR = standardized root-mean-square residual; Δχ^2^ (df) = chi-squared difference test and its degrees of freedom; ΔCFI = change in CFI relative to the preceding model; *p* = *p*-value.

**Table 2:** Model fit statistics for measurement invariance concerning grade level.

Model	χ^2^ (df)	*p*	CFI	TLI	RMSEA	SRMR	Δχ^2^ (df)	ΔCFI	*p*
1a: configural (grade 8)	193.684 (72)	<0.001	0.936	0.919	0.072	0.055	-	-	-
1b: configural (grade 9)	234.493 (72)	<0.001	0.925	0.906	0.075	0.069	-	-	-
1c: configural (grade 10)	177.711 (72)	<0.001	0.953	0.941	0.062	0.060	-	-	-
2: baseline	605.543 (216)	<0.001	0.938	0.922	0.070	0.062	-	-	-
3: metric	634.619 (238)	<0.001	0.937	0.928	0.067	0.067	29.076 (22)	−0.001	0.234
4: scalar	695.407 (260)	<0.001	0.931	0.928	0.067	0.069	60.788 (22)	−0.006	<0.001

χ^2^(df) = chi-squared test of model fit and its degrees of freedom; CFI = comparative fit index; TLI = Tucker–Lewis index; RMSEA = root-mean-square error of approximation; SRMR = standardized root-mean-square residual; Δχ^2^ (df) = chi-squared difference test and its degrees of freedom; ΔCFI = change in CFI relative to the preceding model; *p* = *p*-value.

**Table 3:** Subgroup prevalence rates of school burnout.

Subgroup	n (Total)	n_ddc_ (“Burnt Out”)	Prevalence in % (ddc)	n_crc_ (“Burnt Out”)	Prevalence in % (crc)
Girls	625	143	22.9 *	25	4.0
Boys	479	86	18.0 *	23	4.8
8th graders	326	61	18.7	18	5.5
9th graders	404	82	20.3	15	3.7
10th graders	384	90	23.4	18	4.7
Total	1114	233	20.9	51	4.6

ddc = distribution-dependent criterion; crc = content-related criterion; * denotes a significant difference with *p* < 0.05.

## Data Availability

The raw data supporting the conclusions of this article will be made available by the authors upon request.

## Appendix A

**Original German items of the MBI-SuS**

Subskala Erschöpfung *(subscale exhaustion)*

1. Ich fühle mich von der Schule zermürbt. *(I feel worn down by school.)*
2. Am Ende eines Schultages fühle ich mich erledigt. *(At the end of a school day I feel done.)*
3. Ich fühle mich müde, wenn ich morgens aufstehe und wieder einen Schultag vor mir habe. *(I feel tired when I get up in the morning and have another day of school ahead of me.)*
4. Zur Schule zu gehen ist wirklich eine Belastung für mich. *(Going to school is really a burden for me.)*
5. Durch die Schule fühle ich mich erschöpft. *(School makes me feel exhausted.)*

Subskala Zynismus *(subscale cynicism)*

6. Mein Interesse an den Schulfächern lässt immer mehr nach. *(My interest in school subjects is waning more and more.)*
7. Ich zweifle an der Bedeutung, die die Schule hat. *(I doubt the importance that school has.)*
8. Zur Schule zu gehen bringt doch sowieso nichts. *(Going to school is useless anyway.)*
9. Meine Begeisterung in Bezug auf die Schule hat nachgelassen. *(My enthusiasm in terms of school has waned.)*

Subskala Effizienz *(subscale efficacy)*

10. Ich kann die Probleme, die bei mir in der Schule auftreten, gut lösen. *(I am good at solving the problems that arise with me at school.)*
11. Ich glaube, dass ich zu den Unterrichtsstunden einen wertvollen Beitrag leiste. *(I believe that I make a valuable contribution to the lessons.)*
12. Meiner Meinung nach bin ich ein*e gute*r Schüler*in. *(In my opinion, I am a good student.)*
13. Es motiviert mich, wenn ich meine Ziele in der Schule erreiche. *(It motivates me when I achieve my goals at school.)*
14. [Im Verlauf meiner Schulzeit habe ich viele interessante Dinge gelernt. *(In the course of my school years I learned many interesting things.)*] *
15. In den Unterrichtsstunden bin ich zuversichtlich, dass ich die Dinge erfolgreich bewältige. *(In the lessons, I am confident that I can handle things successfully.)*

* This item was removed as part of the examination of the instrument.
